# Enamel Renal Syndrome: Protocol for a Scoping Review

**DOI:** 10.2196/29702

**Published:** 2021-11-30

**Authors:** Imaan A Roomaney, Salma Kabbashi, Manogari Chetty

**Affiliations:** 1 Department of Craniofacial Biology University of the Western Cape Cape Town South Africa

**Keywords:** enamel renal syndrome, amelogenesis imperfecta, gingival fibromatosis, FAM20A gene, nephrocalcinosis, dentistry, failed tooth eruption, scoping review, dentist, renal evaluation, oral rehabilitation, quality of life, rare conditions, pathophysiology, dental perspective

## Abstract

**Background:**

Enamel renal syndrome (ERS) (OMIM 204690) is a rare autosomal recessive disorder characterized by hypoplastic amelogenesis imperfecta, failed tooth eruption, intrapulpal calcifications, gingival enlargement, and nephrocalcinosis. The rarity of the condition and the variability of the phenotype has led to ERS not being fully characterized.

**Objective:**

This scoping review aims to account for the range and current state of knowledge on ERS and synthesize these findings into a comprehensive summary, focusing on the pathophysiology, genotype-phenotype correlations, and patient management from a dental perspective.

**Methods:**

The authors will conduct a systematic search of PubMed (MEDLINE), BioMed Central, EbscoHost Web, Web of Science, and WorldCat. We will include all studies with human participants with a confirmed diagnosis of ERS. Articles will be screened in two stages (ie, initially by title and abstract screening and then full-text screening by two independent reviewers). Data extraction will be conducted using a customized electronic data extraction form. We will provide a narrative synthesis of the findings from the included studies. We will structure the results according to themes.

**Results:**

This protocol is registered with the Open Science Framework. The electronic search was conducted in July 2020 and updated in April 2021. The research findings will be published in an open access journal.

**Conclusions:**

Dentists should be able to identify patients with clinical features of ERS so that they receive appropriate referrals for renal evaluation, genetic counseling, and oral rehabilitation to increase the patient’s quality of life. A scoping review is the most appropriate method to conduct this comprehensive exploration of the current evidence, which may be sparse due to the rarity of the condition. It will also enable us to identify gaps in the research.

**Trial Registration:**

Open Science Framework; https://osf.io/cghsa

**International Registered Report Identifier (IRRID):**

DERR1-10.2196/29702

## Introduction

Enamel renal syndrome (ERS) (OMIM 204690) is a rare autosomal recessive disorder characterized by hypoplastic amelogenesis imperfecta (AI), failed tooth eruption, intrapulpal calcifications, gingival enlargement, and nephrocalcinosis [1]. In 1972, MacGibbon [2] described a condition presenting with renal dysfunction and enamel hypoplasia in several members of the same family. Although there has been an increasing number of publications about ERS in recent years, the rarity of the condition and the variability of the phenotype has led to ERS not being fully characterized.

Several phenotypes have been identified under various terms, including AI syndrome, AI with interradicular dentine dysplasia, AI with odontogenic fibroma-like hamartomas around nonerupted teeth, AI with gingival fibromatosis [3], MacGibbon syndrome, and Lubinsky-MacGibbon syndrome [4]. The lack of strict diagnostic criteria has led to patient’s renal status being overlooked within those phenotypes, along with underestimation of the actual disease prevalence [3]. This rare genetic entity accounts for less than 1 in 100,000 individuals of the global population, with only 70 cases in 50 different families being documented at present [5].

Recently, high throughput genetic technologies such as whole exome sequencing, next generation sequencing, and bioinformatics analysis implicated mutations in FAM20A (FAMily with sequence similarity 20A) as the defective gene in ‘‘Amelogenesis Imperfecta and Gingival Fibromatosis Syndrome’’ (AIGFS; OMIM 614253). A comprehensive review of AIGFS’s clinical aspect delineated a similar distinctive oral phenotype to ERS [6]. It is believed that both syndromes reflect different phenotypes of the same disease with frequent renal dysfunction association in ERS [3]. More than 40 homozygous or combined heterozygous FAM20A mutations have been identified in families (Human Gene Mutation Database). The protein encoded by the FAM20A gene is secreted by ameloblasts, odontoblasts, gingival, and dental pulp cells, reflecting an important role during enamel development and gingival homeostasis [1,7].

Generally, patients with ERS seek dental management, as the initial chief complaint relates to the lack of enamel and failure of permanent tooth eruption at a young age [6]. A review by de la Dure-Molla and colleagues [3] suggested a distinctive pathognomonic oral profile for patients with ERS (Textbox 1).

In addition to the “common profile” of ERS, several atypical features have been reported in the literature. Pêgo et al [8] reported an association of hypertrichosis and hearing loss in two patients with ERS. Hearing loss is a common feature of Raine syndrome (OMIM 259775), a syndrome caused by FAM20C mutation. This may explain the presence of such a feature in ERS [8]. A few characteristics that are considered atypical manifestations in individuals with ERS have been documented in a study conducted by Dourado et al [1]. These were malocclusion, periodontal disease, supraincisive diastema, and intellectual disability. One study documented a case in which all the permanent teeth had erupted, which is considered highly atypical of ERS [5].

It is clear from the growing list of atypical features of ERS that there is still much that is not known and poorly understood about ERS. This scoping review aims to account for the range and current state of knowledge on ERS. These findings will be synthesized into a comprehensive summary, focusing on the pathophysiology, genotype-phenotype correlations, and patient management from a dental perspective. The authors also aim to elucidate research gaps to inform future research and provide a useful summary for clinicians treating patients with ERS.

**Common oro-dental features of Enamel Renal Syndrome (ERS) according to de la Dure Molla et al [3].**

**Common oro-dental features of ERS**

- Generalized thin hypoplastic or absent enamel
- Primary and permanent teeth affected
- Flat cusps on posterior teeth
- Relative microdontia and spaced teeth
- Intrapulpal calcifications
- Delayed tooth eruption
- Impacted posterior teeth with hyperplastic follicle (hamartoma-like) and altered eruption pathway
- Root dilacerations of impacted teeth
- Gingival fibromatosis (variable severity)
- Gingival and dental follicle ectopic calcification on biopsies
- Semilunar shape of central incisor edge
- Crown resorption of nonerupted teeth
- Anterior open bite
- Root hypercementosis and interradicular dentine dysplasia
- Supernumerary teeth

## Methods

We will undertake this scoping review following the PRISMA-ScR (Preferred Reporting Items for Systematic Reviews and Meta-Analysis Extension for Scoping Reviews) guidelines [9]. This study is registered with the Open Science Framework (OSF) [10].

### Research Questions

The primary research question is “what is known from the existing literature about the oro-dental features of ERS?” The research subquestions are as follows:

- Which methods are used to diagnose ERS?
- What are the clinical and radiographic finding in patients with ERS?
- What are the oral histological findings in patients with ERS?
- What phenotype-genotype correlations have been established?
- What guidelines exist for dentists and dental specialists managing patients with ERS?
- What gaps in the literature exist regarding ERS from the dentist’s perspective?

### Information Sources and Search Strategy

The authors will perform a search of the following online databases: PubMed (MEDLINE), BioMed Central, EbscoHost Web, Web of Science, and WorldCat. Additional studies will be sought using the reference lists of included studies and searching Google Scholar. An example of the search strategy is presented in Table 1. The search will be tailored to each database. The search will be limited to title and abstracts to find studies focused on ERS. No other limiters will be used in the search. References will be managed in Zotero reference manager software (Corporation for Digital Scholarship).

**Table 1:** Example of search strategy to be used in this study (PubMed, Medline; July 23, 2020; limits: none).

No.	Search	Result, n
1	enamel renal syndrome[Title/Abstract] OR FAM20A[Title/Abstract]	52
2	((ENAMEL-RENAL-GINGIVAL SYNDROME[Title/Abstract]) OR (AMELOGENESIS IMPERFECTA, HYPOPLASTIC, WITH NEPHROCALCINOSIS[Title/Abstract])) OR (AMELOGENESIS IMPERFECTA[Title/Abstract] AND GINGIVAL FIBROMATOSIS SYNDROME[Title/Abstract])	19
3	amelogenesis imperfecta type IG[Title/Abstract]	1
4	#1 OR #2 OR #3	60

### Inclusion Criteria

This scoping review will include all research related to our objectives. All primary studies will be included, and review articles will be used for hand-searching of reference lists. No restrictions will be placed on the time frame, language, or gray literature. The population of interest will be limited to human participants (of any age) with a confirmed molecular or clinical diagnosis of ERS. ERS (OMIM 204690) is also known as AI type IG or AI1G, enamel-renal gingival syndrome, AIGFS, and AI with nephrocalcinosis. ERS is caused by homozygous or compound heterozygous mutation in the FAM20A gene (gene number 611062) on chromosome 17q24. The oral clinical features of ERS are considered to be pathognomonic [3]; thus, a clinical diagnosis is considered acceptable for inclusion.

### Study Selection

Articles will be screened in two stages (ie, initially by title and abstract screening, and then full-text screening by two independent reviewers, authors SK and IAR). Any failure of consensus will be resolved by a senior third party (author MC). The degree of agreement between reviewers for each of these steps will be quantified with a kappa statistic with a 95% CI.

### Data Items and Extraction

Data extraction will be conducted by each reviewer independently (IAR and SK). An electronic data extraction form will be custom-made for this review, piloted, and amended as required. The data collection form will include the fields in Textbox 2.

**Data extraction fields.**

**Study information**

- Author(s), title, year of publication, journal
- Study setting
- Study design

**Study population**

- Sample size
- Baseline characteristics, demographics
- Any additional abnormalities or medical conditions
- Any therapies that the patients might have received

**Enamel renal syndrome (ERS)**

- Method of diagnosis
- Family history of the condition
- Genetic variation

**Outcome**

- Prevalence of ERS
- Molecular findings
- Clinical findings (intraoral, extraoral, hematological and other biomarkers, nephrological, other findings)
- Radiographic findings
- Transcriptomic findings of oral tissues
- Dental ultrastructural findings
- Any additional findings

### Synthesis of Results

We will provide a narrative synthesis of the findings from the included studies. We will structure the results according to themes in the following manner to provide a coherent summary of findings: characteristics of included studies (location, study design), baseline characteristics of the participants/population (eg, age and sex), the prevalence of ERS, methods of diagnosis, clinical findings (oral and extraoral), radiographic findings, histological findings, nephrological findings, atypical findings, biomarkers, genotype-phenotype correlation, atypical/novel findings, and additional findings.

## Results

There was no patient participation in this study. This protocol was completed in July 2020 and registered in OSF [10] in August 2020. The electronic search for literature was undertaken in July 2020 and updated in April 2021. The first search yielded 122 titles. This study is intended for open access publication for wide dissemination of the findings.

## Discussion

This scoping review aims to determine the current state of knowledge on ERS and synthesize these findings into a comprehensive summary, focusing on the pathophysiology, genotype-phenotype correlations, and patient management from a dental perspective. We also aim to identify research gaps to inform future research and provide a useful summary for clinicians treating patients with ERS.

ERS is a rare disorder and not much awareness exists around the condition, even within the dental fraternity [11]. Conversely, AI is a relatively common disorder of enamel formation encountered by dentists and often occurs as an isolated trait or part of a syndrome [1]. Because dentists are often the first point of call for these patients, it is important that the dentist is able to identify patients with ERS and differentiate it from AI so that they receive appropriate referrals for renal evaluations and genetic counselling [5]. It is also important to provide the patient with oral rehabilitation to increase the patient’s quality of life, which can be severely impaired by this condition [5]. Primary failure of eruption is difficult to manage, and there is no current literature about assisted eruption with orthodontic traction in patients with ERS [11]. It does appear that the normal eruptive process of teeth is altered leading to impaction and ankylosis [11]. Progressive embedding of the teeth has also been reported [4]. Therefore, orthodontic treatment may be unpredictable. Current management involves surgical interventions and the placement of a fixed or removable dental prosthesis [12,13]. There is a need for guidelines on the dental management of patients with ERS and the development of referral protocols.

Identifying patients with ERS may not be as straightforward as it was previously believed to be. According to de la Dure-Molla et al [3], the oral features of ERS described in Table 1 are considered pathognomonic. However, more recent studies have found significant variability in phenotypes including identifying a patient with a pathogenic variant of the FAM20A gene and a full complement of erupted permanent teeth [5]. Therefore, a review is needed to consolidate this growing body of evidence and present it in a manner that is practical for the dental clinician. We believe that a scoping review is the most appropriate method to conduct a comprehensive exploration of the current evidence, which may be sparse due to the rarity of the condition. It will also enable us to identify gaps in the research.

## Abbreviations

AI: amelogenesis imperfecta
AIGFS: amelogenesis imperfect and gingival fibromatosis syndrome
OSF: Open Science Framework
PRISMA-ScR: Preferred Reporting Items for Systematic Reviews and Meta-Analysis Extension for Scoping Reviews
